# Challenging Childhood Obesity: The Influence of Education and Close Monitoring on Obesity-Related Behaviors

**DOI:** 10.3390/healthcare12202048

**Published:** 2024-10-16

**Authors:** Eda Sunnetci Silistre, Alihan Yesil, Tugba Kozanoglu, Mehmet Cihan Balci, Meryem Karaca, Gulden Fatma Gokcay

**Affiliations:** 1 Department of Child Health and Diseases, Acibadem Kozyatagi Hospital, Istanbul 34734, Türkiye; 2 Department of Pediatrics, Istanbul Faculty of Medicine, Istanbul University, Istanbul 34104, Türkiye; alihan.yesil@istanbul.edu.tr (A.Y.); tugba.saglam@istanbul.edu.tr (T.K.); mcbalci@istanbul.edu.tr (M.C.B.); meryem.karaca@istanbul.edu.tr (M.K.); ghuner@istanbul.edu.tr (G.F.G.)

**Keywords:** childhood, obesity, nutrition education, lifestyle, treatment

## Abstract

Background: We aimed to evaluate the role of nutrition and behavior education intervention in the prevention and treatment of childhood obesity by comparing changes in obesity-related characteristics among obese children during a follow-up period of 12 months. Methods: This study was designed as a prospective cohort study in children aged between 6 and 18 years, with exogenous obesity who applied to Istanbul Research and Training Hospital, Pediatrics Department, between January 2018 and July 2019. Beginning at the sixth month, a program for nutrition and behavior education for obesity prevention and treatment was initiated and continued during the second half of the study period. Results: The mean age of 59 children (29 females, 30 males) was 11.73 ± 2.78. BMI levels did not show a significant difference in the first 6 months, but decreased significantly during the second 6 months of the study. Screen time, fast eating behavior, overeating behavior and food score index scores also demonstrated significant improvements during the intervention period of the study, between 6 and 12 months. Conclusion: It was concluded that nutrition and behavior education for the prevention and treatment of childhood obesity could be a successful intervention with close follow-up.

## 1. Introduction

Obesity can be defined as the accumulation of adipose tissue. It endangers human health in both the short and long term. The World Health Organization (WHO) states obesity as one of the most important health problems in the 21st century. Obesity develops as a result of permanent or longstanding excess of energy intake; that is, when the amount of energy consumed is consistently greater than the amount disposed [1,2]. The etiology of obesity consists of a combination of many factors [3], but dietary habits, domestic life and psychological characteristics have a central role in relation with external factors and biological disposition [4,5]. Consuming obesogenic foods that promote weight gain and limited physical activity are the primary risk factors for obesity [6]. Although the developing child undoubtedly requires some amount of energy excess to facilitate growth, children who develop exogenous obesity due to consuming more calories than necessary are at significantly greater risk of obesity in adult life [7,8].

The ‘obesity epidemic’ has become a grim problem in developed and some developing countries [9]. As such, many leading healthcare organizations (including WHO) and pediatricians recommend comprehensive interventions to reduce childhood obesity [10]. It is widely accepted that interventions to develop a healthy lifestyle during childhood are more effective than interventions in later years of life [11], even though their influence may be limited due to various factors such as sleep time, screen time, fast eating and poor quality diet [12]. Researchers have aggregated data from countless studies on this topic into various systematic reviews focusing on the prevention of childhood obesity [10,13,14]. According to these studies, it is still evident from the plethora of inconsistent data that outcomes of such interventions may be insufficient due to various highlighted reasons, including low efficacy of interventions, differences in study designs and contradictory outcomes of applied measurement methods. Although there are various studies focusing on this topic, there is a great need for further research since the majority of these studies are not based on the assessment of intervention(s).

The objective of our study was to analyze whether childhood obesity could be challenged with educational intervention in a group of children aged 6 to 18 years by comparing the changes in patient characteristics throughout a 6-month period without intervention compared to a 6-month period with nutrition and behavior education and close follow-up.

## 2. Materials and Methods

### 2.1. Study Design and Ethical Issues

This study was designed as prospective cohort study consisting of children with exogenous obesity that were admitted to the Pediatrics Department of Istanbul Research and Training Hospital, between January 2018 and July 2019, for routine pediatric check-up in the presence of complaints associated with obesity, or obesity follow-up.

This study was approved by the local ethics committee and performed after obtaining written informed consent from patients or patients’ parents to participate in this study.

### 2.2. Participants and Study Procedure

Obesity was defined as BMI z-score above +2 standard deviation (SD) with respect to sex and age, and calculations were made using the Antro Program on the WHO website [15]. This study was planned to include 82 school-aged children, aged between 6 and 18 years, diagnosed with exogenous obesity. The plan involved monitoring the group for a total of one year: the first 6 months without any intervention or follow-up, and the second 6 months by providing education on nutrition and a protective lifestyle against obesity, while tracking obesity indicators and adherence to the education program. The education intervention, which will be detailed later on, consisted of information about healthy diet and behavior for obesity prevention.

**Figure 1:**
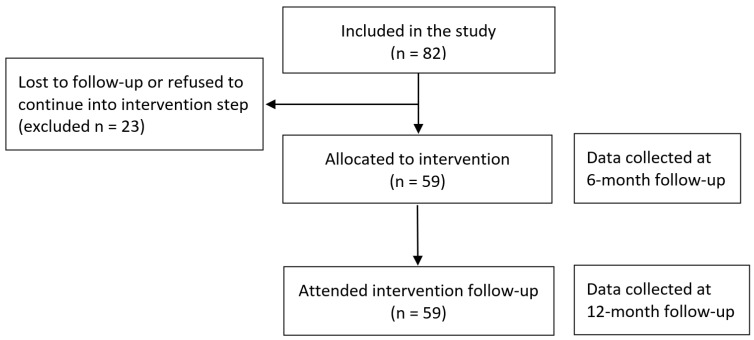
Flow chart of the study.

Children who had been diagnosed with metabolic or syndromic diseases, those that were found to have hormonal abnormalities as a result of routine investigation for obesity causes and those without obesity were excluded from the study. Before enrollment, verbal informed consent was obtained from children and written consent from their families (or legal guardians) for the study. Height, weight and waist circumference measurements of the children were performed by the same pediatrician using the same devices. The same questionnaire was used both at enrollment and at the end of the study. Follow-up procedures of this childhood obesity study were explained and applied by a pediatric specialist to each patient. Additionally, at the 6th month, with the start of the intervention period, the diet and lifestyle information for training prepared according to WHO’s ‘Commission on Ending Childhood Obesity’ (ECHO) program was explained to the child and family by a specialist pediatrician. The study group was initially monitored for six months without any intervention to obtain a baseline and control data, and the measurements were repeated at the end of the six-month period, which was followed by administration of nutrition and behavior education intervention. A flow diagram of the study is shown in Figure 1.

All anthropometric measurements (height, weight and waist circumference) were performed at the beginning of the study and at each monthly interval during the second six months of the study. The height and weight of parents were measured at the first interview to calculate their BMIs. The diet and lifestyle questionnaire was repeated on the 12th month. The aim was to evaluate the effect of the training given in the 6th month, and to assess its reinforcement during monthly controls with respect to anthropometric measurements. Weight was measured with the children wearing underwear by an electronic scale in a room with at least one of the parents present (SECA digital scale with 0.1 g precision and error margin, Hamburg, Germany). Height was measured with the Harpenden stadiometer (Secamod 240, Hamburg, Germany) with an error margin of 0.1 cm. It was decided to make height measurements in a vertical position with bare feet placed in parallel and with the shoulders and the gluteal region in contact with the wall. A non-elastic 7 mm thick SECA measuring tape was used for waist circumference measurements, which were performed from the midpoint between the arcus costae and spina iliaca anterior superior. Results were recorded in centimeter (cm) and waist/height ratio was calculated.

The age of the parents, socioeconomic status, the number of siblings, the education level of parents, monthly income, BMI values, the duration of breastfeeding and the type of delivery were recorded in addition to medical information. The average BMI of parents was also calculated ([mother BMI + father BMI]/2). Additionally, we evaluated sleep time, screen time (time spent in front of a screen), study time, food index score, fast eating behavior and overeating behavior. The number of meals consumed on a daily basis were also recorded at baseline and at each follow-up. These concepts were explained to the participants beforehand. Afterwards, they were asked to base their responses with respect to the period between the previous and the current follow-up. Simple questions were then asked, and the answers were recorded.

The scale for eating habits, developed by Magriplis et al. (2015), was used and the effects of diet on being overweight or obese in school-age children were examined. Foods were organized as positive and negative by their abilities to cause obesity according to available evidence and food guides [16]. A system evaluating nutrition properties (food index score) that was scored between 1 and 4 points for each item was applied, with a higher score associated with non-obesogenic nutrition and a lower score associated with obesogenic nutrition. Possible scores were between 16 and 64 points, and higher scores on the scale indicated a healthier diet in school-age children.

### 2.3. Education Program

For the first six months, only weight, height and waist circumference measures were recorded, and nutrition and behavior education interventions were not applied. Baseline anthropometric data obtained throughout this period were accepted in the form of a set of ‘control’ data for the study group. A structured education program consisting of 10 main goals was initiated in all patients at the end of the 6th month. The education included knowledge of nutrition, healthy foods, food groups, daily nutrients and unhealthy diet models that should be avoided. No written diet or prescribed menu were given to the patients during the study, and no nutrient analyses that evaluated nutrient consumption were applied. From the 6th to the 12th month, the patients’ compliance with the education was evaluated verbally on a monthly basis, and in each evaluation, the information was repeated with respect to the level of knowledge of the patient. Education was maintained until the patient had complete awareness of the information provided to them, regardless of whether they were actually practicing. The children also filled out a monthly motivation assessment questionnaire according to the WHO ECHO program recommendations to assess the 10 main educational goals.

The training was carried out by a specialist pediatrician in line with WHO ECHO recommendations by explaining face-to-face on paper. The duration of the education was 45 min.

Training topics are listed below.

- Healthy nutrition;
- Food groups;
- Portioning;
- Foods that should be increased in consumption;
- Obesity;
- Management of obesity and body weight;
- Obesity and nutrition;
- Physical activity goals;
- Screen time;
- Sleep duration.

The training goals were as follows:

1. To have breakfast every day;
2. Drink 8 glasses of water every day;
3. Consume half of the amount of grains they eat as whole grains;
4. Consume 3 portions of vegetables and 4 portions of fruit every day;
5. One hour of light exercise every day and 30 min of vigorous exercise 3 days a week;
6. Total time in front of the screen should not exceed 2 h;
7. Sleep duration above 7–8 h.

Before these objectives were explained, the following concepts were explained to the children

- Food groups;
- Portioning;
- Fruits, vegetables;
- What does whole grain mean;
- What are light exercises, how long should they be;
- What are heavy exercises, how long should they be;
- What is screen time, and how long should it be.

At 6-7-8-9-10-11-12 months, the children and the parents were scheduled for follow-up visits. Education was repeated and anthropometric measurements were made. There was no dietary program and food consumption record. A motivation book was given to the individuals. The purpose of the motivation book was to remind and motivate, not to evaluate.

The motivation book contained the following items:

- Did you have breakfast today?
- How many glasses of water did you drink today?
- Did you drink sugary fizzy drinks today?
- How many portions of vegetables did you eat today?
- How many servings of fruit did you consume today?
- How many servings of whole grain foods did you eat today?
- Did you do light or heavy exercise today?
- How many hours did you spend in front of a screen today?
- How many hours of sleep did you get today?

### 2.4. Statistical Analysis

The SPSS 23.0 and STATA 14.0 programs were used for data analysis. Descriptive statistics (mean, standard deviation, median and range as min–max), histogram and boxplot graphs were used to assess distribution of variables and to depict the resultant data. The Wilcoxon or Friedman tests were used to investigate the differences between measurements that were not distributed normally. The paired sample t-test (between groups) or ANOVA (time-bound comparisons) were used to assess normally distributed data. A marginal homogeneity test was used to evaluate categorical variables. In the presence of p-values of 0.05 or lower, we accepted that the tests showed statistically significant results.

## 3. Results

An initial number of 82 children with exogenous obesity (z-score above +2 standard deviation) were enrolled in the study from January 2018 to July 2019. Twenty-three patients who did not attend their follow-up examinations were excluded, and analysis was performed on 59 patients. Mean age and standard deviation of the study group was 11.73 ± 2.78 years. The study group comprised 29 females (49.2%) and 30 males (50.8%) (Table 1).

**Figure 2:**
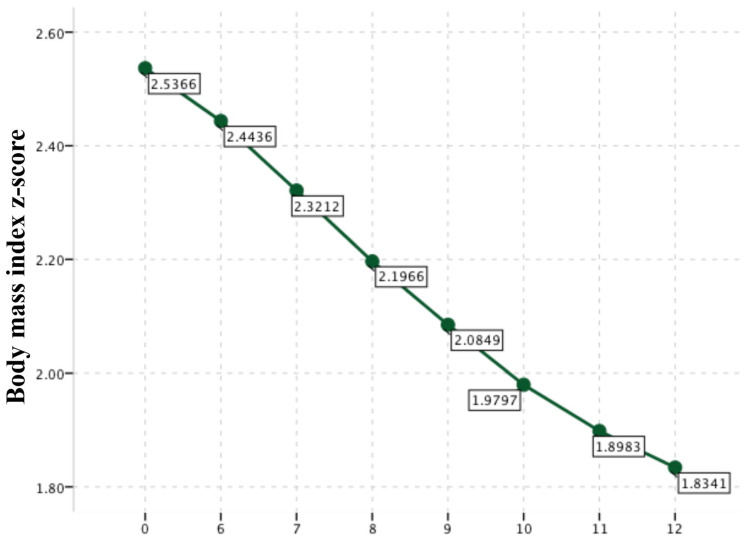
BMI z-scores of the study group (n = 59), during the study period of 12 months.

There was no significant difference in BMI values during the first 6 months, but there was a statistically significant decrease when final BMI was compared to the initial and 6th month assessments (*p* < 0.001 for both comparisons). There was no significant difference in waist circumference values during the first 6 months, but there was a statistically significant decrease when final waist circumference was compared to the initial and 6th month assessments (*p* < 0.001 for both comparisons) (Table 2). BMI z-score of the study group (n = 59) during the study period of 12 months are shown in Figure 2.

Increased study time and decreased screen time were observed at the end of the study compared to the 6th month analysis (*p* = 0.002 and *p* = 0.049, respectively). Fast eating behavior and overeating behavior decreased significantly after intervention (*p* < 0.001 for both). Increase in food index score after intervention was also significant (*p* < 0.001) (Table 3).

## 4. Discussion

This study aimed to evaluate whether changes in obesity-related parameters could be obtained in a group of Turkish children with obesity who received nutrition and behavior education intervention for the prevention and treatment of exogenous obesity, with comparisons performed with respect to a baseline control period of 6 months without any intervention. It is evident from this data that, in addition to positive effects on BMI z-scores, education and close follow-up activities can be helpful in gaining positive nutritional behaviors. Statistically significant changes in food index score and other healthy behaviors were achieved with education, demonstrating that successful outcomes can be obtained by providing education without limiting children to a prescribed diet that will be inefficient for the sustainability of obesity control after intervention period.

Health education is a direct method helping the development health literacy, which includes the ability to understand health information and to make rational decisions about personal health [17]. Health education aims to elicit healthy eating habits in childhood with the likelihood of being maintained throughout the whole life, thereby enabling the avoidance of obesity [18]. These kinds of interventions seek to achieve changes in attitudes and behaviors, while also facilitating objective improvements, such as decreased BMI [19]. In our study, BMI scores decreased in the intervention period, whereas the pre-intervention control period indicated no significant change. In addition to reduced BMI, positive changes were observed in screen time, food index score, fast eating and overeating. In a systematic review, it was found that nutritional education had a serious impact on reducing obesity [20], possibly through increased awareness of nutritional behavior [21]. It was also shown that such interventions resulted in decreased BMI z-scores similar to our results, and positive changes in waist circumference were obtained in various studies [22]. Similar outcomes are reported in other studies demonstrating relationships between nutrition, physical activity, healthy lifestyle and obesity [23,24,25,26,27,28,29,30,31,32,33]. Additionally, since food index scores increased positively in our study, it is evident that the increase in knowledge translated into avoidance of obesogenic foods, which, in turn, can prevent or treat obesity in children.

We also examined other characteristics, such as sleep duration, which is an indispensable part of life and can be associated with obesity. It is acknowledged that there is relationship between shortening of sleep time and obesity due to increase in the frequency of eating [34]. There was no statistically significant difference between the sleep times of participants in the comparison of pre- and during-intervention analyses in our study. This result is thought to be related to the fact that participants’ sleep times were within acceptable limits at baseline, and remained consistent throughout the study period. In addition, we observed that the number of meals consumed daily were similar before and after training. In a national study, it was found that 36.8% of obese and 23.8% of normal weight children ate at least one of three daily meals irregularly, the majority of which were skipping meals [35]. In another national study in which the nutritional habits and obesity states of 425 students were examined, the authors found that 68.5% of students were consuming three meals a day, and the most commonly skipped meal was breakfast (47.3%) [36]. Our findings were compatible with the literature.

In the current study, the frequency of fast eating behavior decreased significantly after nutrition and behavior education intervention. Fast eating and less chewing are among the factors that are closely associated with obesity development [37], since it has been demonstrated that obesity is more common in persons with fast eating behavior [38,39]. There was a significant decrease in screen time after intervention (*p* = 0.049). Studies have reported that obesity is directly linked to the presence of a TV in the child’s room [40,41]. It is now well-established that, as screen time increases, BMI and prevalence of obesity increases [42,43]. In fact, a remarkable study reported that an hour of increase in screen time per day resulted in a 7% increase in the likelihood of adulthood obesity [43]. When classification was made according to BMI, it was reported that increased screen time was directly associated with the risk of being overweight or obese [44]. According to these results and our findings, it appears that it will be beneficial to incorporate education about screen time, sleep and fast eating habits into nutritional intervention measures aimed at preventing and treating obesity.

Despite reporting interesting data pertaining to the utility of educational training in Turkish children with obesity, our study has various limitations. First, this was not a randomized study, and only subjects who could attend the follow-up studies regularly and agreed to participate in the study were enrolled. The likelihood of attending these studies may be associated with sociodemographic characteristics and economic status [45]. The awareness and economic status of parents who continued to attend follow-up studies regularly may have been better compared to those who were lost to follow-up and excluded from the study. Second, we did not examine external factors that could aid the development of healthy lifestyle habits and their role in the increased awareness of children and/or their parents. Therefore, our cohort may have been composed of subjects who had prior awareness concerning this topic, and therefore, had applied with greater motivation to receive support to prevent obesity. Although this is a limitation, it also indicates that nationwide programs should be undertaken to at least create awareness that pediatric follow-up for obesity is an important approach. Third, in relation with the last point, it is possible that the subjects recruited to the study were also obtaining obesity-related knowledge from other sources, but we directly associated the post-interventional changes to the education given throughout the study. Nonetheless, since these patients did not show significant BMI change during the initial 6 months of the study, it is highly likely that positive effects were indeed a result of the education provided during the study at our center. The parents of almost half of the participants had only elementary school education, and the majority had an income of either minimum wage or up to double minimum wage. This may have affected the results, especially with respect to access to non-obesogenic nutrition. Admittedly, the cohort was self-selected, since 28% of the participants did not continue into the intervention period. Furthermore, this was only a 6-month intervention, a period when weight regain typically occurs after such interventions. These limitations should be considered when interpreting the results of this study. Additionally, this study deviated from the standard educational method, in that we continued to provide training throughout the study period and performed close monitoring, which would have increased the likelihood of positive outcomes compared to standard training interventions.

## 5. Conclusions

There was a significant difference in BMI values of participants over the 6 months after nutrition and behavior education intervention, while no significant change was observed in the initial 6-month control follow-up period. In addition to this highly quantifiable improvement, we reported progress in nutritional behaviors, screen time and healthy nutrition knowledge. It is necessary to confirm these results in more extensive studies and in other populations to ascertain whether educational training can be an easily applicable, cheap and reliable instrument in the prevention and treatment of childhood obesity.

## Figures and Tables

**Table 1:** Demographic data of the study group.

	n	Mean ± SD	Range (Min–Max)
**Birth Weight (g)**	59	3319.58 ± 523.56	2250–4750
**Breastfeeding (month)**	59	16.03 ± 9.30	0–36
**Mother BMI (kg/m^2^)**	59	28.12 ± 3.87	22.1–38.5
**Father BMI (kg/m^2^)**	59	29.30 ± 4.09	18.7–38.5
**Parents BMI (kg/m^2^)**	59	28.71 ± 3.05	23.30–35.6
	**n**	**Ratio (%)**	
**Delivery (birth)**			
Normal	35	59.3	
C-section	24	40.7	
**Education Status of Mother**			
Elementary	32	54.2	
Secondary	10	16.9	
High school	16	27.1	
University	1	1.7	
**Education Status of Father**			
Elementary	25	42.4	
Secondary	13	22.0	
High school	19	32.2	
University	2	3.4	
**Level of income**			
Minimum wage (around 300 US dollars)	24	40.6	
Up to double minimum wage	29	49.2	
≥Minimum wage × 3	6	10.2	
**Parents’ BMI Values**			
Normal (parents BMI < 25)	6	10.2	
Overweight (≥25 parents BMI < 30)	35	59.3	
Obese (≥30 parents BMI < 35)	16	27.1	
Morbid obese (parents BMI ≥ 35)	2	3.4	

BMI: body mass index, SD: standard deviation, parent BMI = (mother BMI + father BMI)/2.

**Table 2:** Comparison of first, 6th month and 12th month BMIs of the study group (n = 59).

Measurement Period	Month	BMI (Mean ± SD) (kg/m²)	*p*
0–6 months (pre-education)	0	28.73 ± 3.57	0.775
	6	28.68 ± 3.37	
6–12 months (post-intervention)	6	28.68 ± 3.37	0.001
	12	25.98 ± 3.46	
0–12 months	0	28.73 ± 3.57	0.001
	12	25.98 ± 3.46	
		**Waist circumference (cm)**	
0–6 months (pre-education)	0	94.34 ± 11.74	0.761
	6	94.50 ± 11.79	
6–12 months (post-intervention)	6	94.50 ± 11.79	0.001
	12	92.76 ± 11.06	
0–12 months	0	94.34 ± 11.74	0.001
	12	92.76 ± 11.06	

SD: standard deviation, *p* < 0.05: statistically significant.

**Table 3:** Comparison of screen time, sleep time and study time before and after the education of the study group (n = 59).

	Month	Duration (Mean ± SD) (Hours)	Number of Cases, n (%)	*p*
Sleep Time	6	7.89 ± 0.75		1.000
	12	7.89 ± 0.75		
Screen Time	6	3.23 ± 1.22		0.049
	12	3.08 ± 1.14		
Study Time	6	1.33 ± 0.54		0.002
	12	1.49 ± 0.62		
Food index score	6	33.76 ± 4.39		0.001
	12	43.19 ± 5.32		
Fast eating	6		45 (76.3)	0.001
	12		27 (45.8)	
Overeating	6		53 (89.8)	0.001
	12		33 (55.9)	
Number of meals daily (4 or over)	6		33 (55.9)	1.000
	12		33 (55.9)	

SD: standard deviation, *p* < 0.05: statistically significant.

## Data Availability

The data presented in this study are available on request from the corresponding author.
